# Age-Related Unstructured Spike Patterns and Molecular Localization in *Drosophila* Circadian Neurons

**DOI:** 10.3389/fphys.2022.845236

**Published:** 2022-03-09

**Authors:** Dieu Linh Nguyen, Anelise N. Hutson, Yutian Zhang, Skylar D. Daniels, Aidan R. Peard, Masashi Tabuchi

**Affiliations:** Department of Neurosciences, Case Western Reserve University School of Medicine, Cleveland, OH, United States

**Keywords:** *Drosophila*, circadian clock, sleep, aging, membrane potential, electrophysiology, DN1p

## Abstract

Aging decreases sleep quality by disrupting the molecular machinery that regulates the circadian rhythm. However, we do not fully understand the mechanism that underlies this process. In *Drosophila*, sleep quality is regulated by precisely timed patterns of spontaneous firing activity in posterior DN1 (DN1p) circadian clock neurons. How aging affects the physiological function of DN1p neurons is unknown. In this study, we found that aging altered functional parameters related to neural excitability and disrupted patterned spike sequences in DN1p neurons during nighttime. We also characterized age-associated changes in intrinsic membrane properties related to spike frequency adaptations and synaptic properties, which may account for the unstructured spike patterns in aged DN1p neurons. Because Slowpoke binding protein (SLOB) and the Na^+^/K^+^ ATPase β subunit (NaKβ) regulate clock-dependent spiking patterns in circadian networks, we compared the subcellular organization of these factors between young and aged DN1p neurons. Young DN1p neurons showed circadian cycling of HA-tagged SLOB and myc-tagged NaKβ targeting the plasma membrane, whereas aged DN1p neurons showed significantly disrupted subcellular localization patterns of both factors. The distribution of SLOB and NaKβ signals also showed greater variability in young vs. aged DN1p neurons, suggesting aging leads to a loss of actively formed heterogeneity for these factors. These findings showed that aging disrupts precisely structured molecular patterns that regulate structured neural activity in the circadian network, leading to age-associated declines in sleep quality. Thus, it is possible to speculate that a recovery of unstructured neural activity in aging clock neurons could help to rescue age-related poor sleep quality.

## Introduction

Aging influences many physiological processes, including sleep (Bliwise, 1993). In humans, age-dependent declines in the physiological function control the circadian and homeostatic regulation of sleep (Cajochen et al., 2006; Pace-Schott and Spencer, 2011). However, we do not fully understand how aging influences clocks to regulate sleep. The molecular mechanism of circadian clock systems changes with age, reducing sleep quality (Hofman and Swaab, 2006; Kondratov, 2007; Hood and Amir, 2017). Importantly, aging influences the circadian clock neuronal activity patterns (Nakamura et al., 2015, 2016). This mechanism could underlie how aging clocks regulate sleep.

A number of studies in *Drosophila* and mammals examined how aging, from molecules to behavior, affects the circadian clock machinery (Kondratova and Kondratov, 2012). In mammals, the circadian clock network shows age-related electrophysiological changes, including membrane currents and spiking activity, in the suprachiasmatic nucleus (SCN) (Biello, 2009; Farajnia et al., 2014; Buijink and Michel, 2021). However, the mechanistic relationships between core-clock molecular signaling and electrophysiological function are unclear. In *Drosophila*, the aging effects of clock and sleep have been characterized (Koh et al., 2006), and the circadian clock neuronal circuits have been comprehensively studied, so we have a clearer understanding of the causal role of clock neurons in circadian regulation of sleep quality. Based on growing evidence, the neural activity of circadian clock networks that mediate circadian regulation of sleep quality is universal across species, from *Drosophila* to humans. As in mammals, sleep in *Drosophila* is regulated by the circadian clock and homeostatic processes. Thus, to understand age-dependent circadian regulation of sleep, we must uncover how age-associated changes in molecular clock dynamics affect neural activity.

The molecular clock that generates the circadian rhythm is based on a transcriptional-translational feedback loop in *Drosophila* (Rosato et al., 2006; Tataroglu and Emery, 2015). In this loop, studies using *Drosophila* as a model have revealed that two transcriptional activators (CLOCK and CYCLE) drive the expression of two repressors (PERIOD and TIMELESS) that, in turn, bind and inhibit CLOCK/CYCLE (Hardin et al., 1990; Sehgal et al., 1995; Allada et al., 1998). Later on, such negative feedback loops have been found to be essentially conserved in mammals, although some specific molecular components show differences between *Drosophila* and mammalian circadian clocks (Ko and Takahashi, 2006; Duong et al., 2011; Partch et al., 2014).

A major contributor to circadian networks is posterior DN1 (DN1p) circadian clock neurons, which regulate sleep/arousal states (Kunst et al., 2014; Guo et al., 2016, 2018; Lamaze et al., 2018; Lamaze and Stanewsky, 2019; Tabuchi et al., 2021). DN1p clock neurons have circadian cycling in their intrinsic membrane properties, which are regulated by clock output molecules such as the sodium leak channel narrow abdomen (Flourakis et al., 2015). The DN1p spiking pattern is irregular during the mid-day (Zeitgeber time of 6-8, ZT6-8) and regular at mid-night (ZT18-20). Importantly, these patterns are associated with differences in the quality, but not the quantity, of sleep and are regulated by two “pattern generators,” Ca^2+^-dependent K^+^-channel binding protein (Slowpoke binding protein, SLOB) and Na^+^/K^+^ ATPase β subunit (NaKβ). These generators are upregulated at night under CLOCK and WAKE control (Tabuchi et al., 2018).

During periods of increased input, upregulated Slowpoke activity leads to a deeper afterhyperpolarization (AHP) of DN1p spikes. Conversely, during periods of reduced input, Na^+^/K^+^ ATPase activity accelerates spike onset, which maintains spiking. This combination of increased KCa and Na^+^/K^+^ ATPase activity promotes spike kinetics with faster onset and deeper AHPs, leading to regular firing and greater sleep quality at night. Based on previous work, we expect that these regulatory functions in DN1p activity patterns decline with age, resulting in unstructured sleep architecture. In addition to intrinsic membrane properties, we also expect that age-related changes in synaptic inputs to DN1ps as other studies using different systems show age-related synaptic alterations (Martinez et al., 2007; Omelyanchuk et al., 2015; Rozycka and Liguz-Lecznar, 2017; Banerjee et al., 2021), and biophysical synaptic drives act to shape persistent spiking activity in general (Zylberberg and Strowbridge, 2017).

In this study, by comparing electrophysiological properties of DN1p neurons between young and aged flies, we showed that spike patterns generated by the clock become enormously unstructured with aging. Moreover, by comparing circadian cycling of subcellular organization between young and aged flies, we demonstrated that such altered spiking patterns are influenced by disrupted SLOB-HA and NaKβ-myc patterns in DN1p neurons. These data showed that aging diminishes tightly controlled molecular organizations that achieve precisely structured activity patterns in the circadian network, leading to age-associated decreased sleep quality.

## Materials and Methods

### Fly Strains

Flies were fed standard *Drosophila* food containing molasses, cornmeal, and yeast. They were housed in a 25°C incubator (DR-36VL, Percival Scientific, Perry, IA, United States) under 12 h:12 h light-dark cycles and 65% humidity. To target DN1p neurons, the wake-Gal4 line was used (Liu et al., 2014). To visualize localization of SLOB and NaKβ, UAS-SLOB-HA and UAS-NaKβ-myc lines were used (Tabuchi et al., 2018). For electrophysiological and immunocytochemical experiments, newly eclosed adult wake-Gal4 > UAS-CD8::GFP, > UAS-SLOB-HA, or > UAS-NaKβ-myc female flies were collected and transferred to food vials at a density of ∼10 flies/vial and maintained at 25 °C under a 12 h:12 h light-dark cycle with 65% humidity. Flies were transferred into fresh food vials every 2 days. Flies that were 2–4 days old were considered “young,” and flies that were approximately 2 months old (60–67 days old) were considered “aged.”

### Electrophysiological Recordings

We conducted electrophysiological recordings from DN1p neurons with *ex vivo* configuration (i.e., isolated brain preparation). Flies were anesthetized by chilling on ice (up to 10 min). Their heads were then isolated and placed in a dissecting chamber. Brains were removed and dissected in a *Drosophila* physiological saline solution (101 mM NaCl, 3 mM KCl, 1 mM CaCl_2_, 4 mM MgCl_2_, 1.25 mM NaH_2_PO_4_, 20.7 mM NaHCO_3_, and 5 mM glucose; pH 7.2) pre-bubbled with 95% O_2_ and 5% CO_2_. To increase the likelihood of successful recordings, brains were treated with an enzymatic cocktail of collagenase (0.1 mg/mL), protease XIV (0.2 mg/mL), and dispase (0.3 mg/mL) at 22°C for 1 min. Then the glial sheath surrounding the brain was focally and carefully removed using sharp forceps. The surface of the cell body was cleaned with a small stream of saline pressure-ejected from a large-diameter pipette under visualization of a dissecting microscope. DN1p neurons were visualized with GFP fluorescence with a PE300 CoolLED illumination system (CoolLED Ltd., Andover, United Kingdom) on a fixed-stage upright microscope (BX51WI; Olympus, Japan). One neuron per brain was recorded.

### Perforated Patch-Clamp Recordings

Perforated patch-clamp recordings of DN1p neurons were performed as described (Tabuchi et al., 2018). Patch pipettes (9–12 MΩ) for perforated patch-clamp were fashioned from borosilicate glass capillary (without filament) using a Flaming-Brown puller (P-97, Sutter Instrument) and further polished with a microforge (MF200, WPI) before filling the internal pipette solution with 102 mM potassium gluconate, 0.085 mM CaCl_2_, 0.94 mM EGTA, 8.5 mM HEPES, 4 mM Mg-ATP, 0.5 mM Na-GTP, 17 mM NaCl, pH7.2. Escin (Santa Cruz Biotechnology) was prepared as a 50 mM stock solution in water (stored up to 2 weeks at −20°C) and added fresh into the internal pipette solution to a final concentration of 50 μM. Due to the lightsensitivity of escin, filling syringes were wrapped with aluminum foil. Pipette tips were dipped into a small container with escin-free internal pipette solution for approximately 1 s, and then backfilled with the escin-containing solution from the filling syringe. Air bubbles were removed by gentle tapping. Escin pipette solutions remained stable for several hours after mixing in the filling syringe, with no evidence of precipitation. Junction potentials were nullified, a high-resistance seal was formed, and perforated patches were allowed to develop spontaneously over time. After breakthrough was evident (based on the gradual development of a large capacitance transient in the seal test window), access resistance was first monitored with the membrane test function and then continuously during the final steps of the perforation process until it became stable (access resistance stably < 40 MΩ). Cells that showed signs of “mechanical” break-in (i.e., a significant increase of time constant of transient capacitive current) were excluded from further data acquisition. During the recording, the bath solution was continuously perfused with saline with a gravity-driven system. Recordings were acquired with an Axopatch 1D, 200A, or 200B amplifier (Molecular Devices) and sampled with PCIe-6341 interface (National Instrument) controlled by Wavesurfer^1^ software (for Axopatch 1D) or Digidata 1550B (Molecular Devices) controlled by pCLAMP 11 (Molecular Devices). The voltage signals were sampled at 10 kHz and low-pass filtered at 1 kHz.

### Intracellular Recordings

To clearly dissociate action potential spikes from postsynaptic potentials (PSPs), sharp electrode intracellular recordings of DN1p neurons were performed as described (Liu et al., 2017). Sharp electrodes from quartz glass with a filament (OD/ID: 1.2/0.6 mm) were fabricated with a laser-based micropipette puller (P-2000, Sutter instrument) and backfilled with 1 M KCl, with resistances of 120–190 MΩ. Solutions were filtered using a 0.02-μm syringe filter (Anotop 10, Whatman). We inserted an electrode into the cell body of DN1p neurons expressing GFP. Impalements of the intracellular electrode were induced using the shortest “buzz” pulses and only buzzing when the electrode was not moving. Because stabilizing the cell membrane potential takes at least 1 min, recordings of membrane potentials began after the cell membrane potential was stable. To separately measure PSPs, the tonic hyperpolarizing current was injected into the targeted cell. We aimed to keep the somatic membrane potential between –105 and −110 mV. The measured PSPs were likely a mix of excitatory and inhibitory synaptic events because targeted cells were hyperpolarized to values near or beyond the reversal potential for inhibitory synaptic currents (up to −110 mV at somatic observation based on the lack of hyperpolarizing PSPs). Recordings were acquired with an Axoclamp 2B with HS-2A × 1 LU headstage (Molecular Devices) and sampled with Digidata 1550B interface, both of which were controlled on a computer using pCLAMP 11 software. The signals were sampled at 10 kHz and low-pass filtered at 1 kHz.

### Analysis of Electrophysiology Data

Electrophysiological analysis was performed in MATLAB (MathWorks). To quantify spontaneous firing activity, we used the coefficient of variation (CV) of interspike intervals (ISI), a global measure of irregularity defined as the dispersion of the ISIs (Holt et al., 1996). We also calculated the local variation (LV) as alternative measures of local irregularity by computing the dispersion of the two adjacent ISIs. LV is defined as


$$L\,v=\frac{1}{n-1}\,\sum_{i=1}^{n-1}\frac{3\,{\left(I\,S\,I_{i}-I\,S\,I_{i\,1}\right)}^{2}}{{\left(I\,S\,I_{i}+I\,S\,I_{i\,1}\right)}^{2}}$$

where *ISI*_*i*_ is the *ith* ISI and n is the number of ISIs (Shinomoto et al., 2003). To assess the shape of the ISI distribution, we calculated skewness and kurtosis based on the histograms. Skewness indicated the symmetry of the ISI distribution around the mean, and kurtosis mirrored the peak of the ISI histogram. To quantify evoked spiking activity, spikes were elicited in response to current injections with 300-ms stepping pulses at 5-pA increments up to 35 pA. The *f-I* curve was computed by sorting the level of injected current. The current threshold (minimal current to evoke spiking) and the slope of the *f-I* curve were determined by linear regression of the curve from the point of initial spiking. Input resistance was calculated from the voltage change obtained by injecting a hyperpolarizing current of 10 pA. Degrees of spike frequency adaptation were assessed by plotting the distribution of instantaneous spike frequency during the depolarizing current injection. Half-life and plateau variables of the process curve of spike frequency adaptation were determined by fitting with a monoexponential function. To detect and quantify PSPs, a median filter with a time constant of 3 ms was applied to the unfiltered membrane potential. Background noise power was computed based on root mean square values from the all-points amplitude in each data set. These computations were used to define both event-finding and noise-rejection criteria, which consists of a minimum-allowed amplitude. To manually check computed results, we visualized all detected PSP events and their peak and quantified amplitudes. We sorted individual PSP epochs and defined their amplitude as the difference between the maximum/minimum potential of each PSP and the mean membrane potential of the entire trace. The relative rising slope of the PSP was calculated as the change in voltage amplitude per millisecond. To estimate the temporal structure of a state transition between miniature PSP (mPSP) and spike-induced PSP (PSP), discrete-time Markov chain was used. We first classified the estimated mPSP and PSP with the k-means clustering algorithm (Dorgans et al., 2019), which was based on average PSP amplitude of each dataset. We confirmed that the separation is a binary distribution by estimating that mPSP has a smaller amplitude and PSP has a larger amplitude (Wierenga and Wadman, 1999). We then created a transition matrix to calculate transition probability.

### Immunocytochemistry

Brains were dissected in a *Drosophila* physiological saline solution, fixed with 4% paraformaldehyde in phosphate-buffered saline (PBS) for 30 min at room temperature, and then washed in PBS. To improve the penetration of antibodies when staining brains, the glial sheath enveloping the brain was carefully removed. Samples were incubated with rat anti-HA at 1:100 (3F10, Roche) or mouse anti-MYC (9E10, Sigma-Aldrich) at 1:50 on a shaker at 4°C for 48 h. After washing 3 times with PBS + 0.1% Tween 20 for 15 min each, samples were then incubated with Alexa Fluor 488-conjugated anti-rat (Invitrogen, 1:1,000) for SLOB-HA, or Alexa Fluor 488-conjugated anti-mouse (Invitrogen, 1:1,000) for NaKβ-myc on a shaker at 4°C for 48 h. After washing 3 times with PBS + 0.1% Tween 20 for 15 min each, samples were cleared in 70, 80, 90, and 100% glycerol in PBS for 5 min at room temperature. Samples were then mounted with a coverslip and Vectashield mounting medium (H−1000, Vector Laboratories). Images were taken under a 100 × magnification objective lens using a Leica TCS SP8 gated stimulated emission depletion 3X system (Leica Microsystems) and acquired as 1,024 × 1,024 pixels (16 bit). A slice having maximum nuclear diameter was used to quantify the appropriate region of interest from each cell. After the acquisition, images were preprocessed by the iterative deconvolution algorithm of the Huygens Professional deconvolution package (Scientific Volume Imaging, Netherlands). Fiji (ImageJ) was used to quantify the intensity of the total, plasma membrane, and perinuclear signals. Background intensity adjacent to the region of interest was measured and subtracted.

### Statistical Analyses

Statistical analyses were performed using Prism software (GraphPad, version 9.3.1.). To compare two groups of data, normally distributed data were compared with *t*-tests, and non-normally distributed data were compared with Mann–Whitney *U*-tests. To compare more than two multiple-group comparisons, two-way ANOVA with multiple comparisons was used. A *p*-value < 0.05 was considered statistically significant, with asterisks indicating *p*-values as follows: *p < 0.05, ^**^p < 0.01, ^***^p < 0.001, and ^****^p < 0.0001. ns indicates non-significance. All error bars represent means ± SEM averaged across experiments.

## Results

### Age Is Associated With Electrophysiological Changes in DN1p Neurons

**FIGURE 1:**
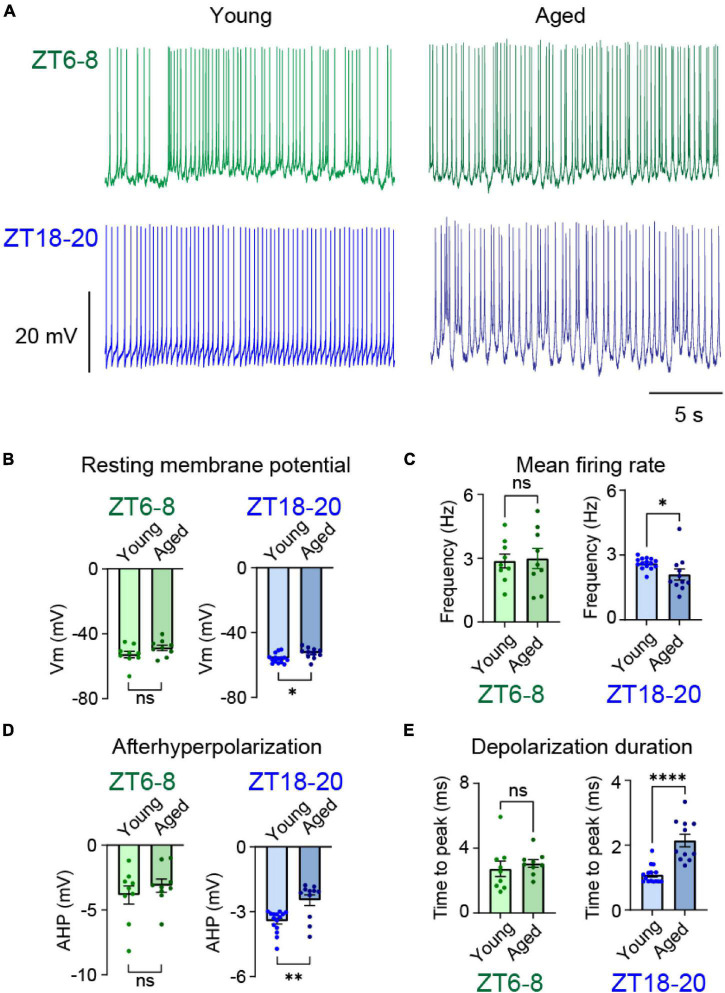
Aging altered the membrane potential dynamics of DN1p neurons at ZT18-20. **(A)** Representative membrane potential traces of spontaneous firing in young and aged DN1p neurons at ZT6–8 and ZT18–20. **(B–E)** Quantifications of resting membrane potential **(B)**, mean firing rate **(C)**, afterhyperpolarization amplitude **(D)**, and spike onset depolarization duration defined as time from threshold to peak depolarization **(E)** in young and aged DN1p neurons at ZT6–8 and ZT18–20 (ZT6–8, young: *n* = 9, aged: *n* = 9; ZT18–20, young: *n* = 15, aged: *n* = 11). *p < 0.05, ^**^p < 0.01, and ^****^p < 0.0001 based on *t*-tests.

The molecular clock dynamically modulates the neural activities of clock networks to regulate circadian physiology and behavior (Allen et al., 2017). Age-related electrophysiological changes in clock neural networks occur in both *Drosophila* and mammals (Watanabe et al., 1995; Curran et al., 2019). However, most research has focused on the clock neuron firing rate, and we do not know if aging influences the temporal structural pattern of clock neuron firing. We focused on posterior DN1 (DN1p) neurons because of their contributions to sleep regulation. Moreover, specific temporal patterns of spontaneous firing activity in DN1p neurons are different at midday (ZT6–8, Zeitgeber Time 6–8) and mid-night (ZT18–20). Thus, they differentially affect sleep architecture, even if the mean firing rate is unchanged. Thus, we compared spontaneous activity in DN1p neurons between young and aged flies. In young flies (2–4 days old), DN1p neurons exhibited irregular firing at ZT6–8 but regular firing at ZT18–20 (Figure 1A). These observations are consistent with another report that used a slightly different age range (4–8 days old). In contrast to young flies, aged flies (60–67 days old) appeared to exhibit similar firing at ZT6–8 and ZT18–20 (Figure 1A).

To identify biophysical parameters underlying age-associated electrophysiological changes, we quantified intrinsic membrane properties during spontaneous activities. We found that the resting membrane potential (Figure 1B), mean firing rate (Figure 1C), spike waveform kinetics (e.g., afterhyperpolarization) (Figure 1D), and spike onset depolarization duration (time from threshold to peak depolarization) (Figure 1E) significantly differed between young and aged DN1p neurons at ZT18–20, but not at ZT6-8. These results suggest that age-related differences in DN1p activity patterns may be due, in part, to changes in intrinsic membrane properties at ZT18–20 rather than ZT6–8.

### Age Is Associated With Unstructured Spike Patterns of Spontaneous Activity in DN1p Neurons

**FIGURE 2:**
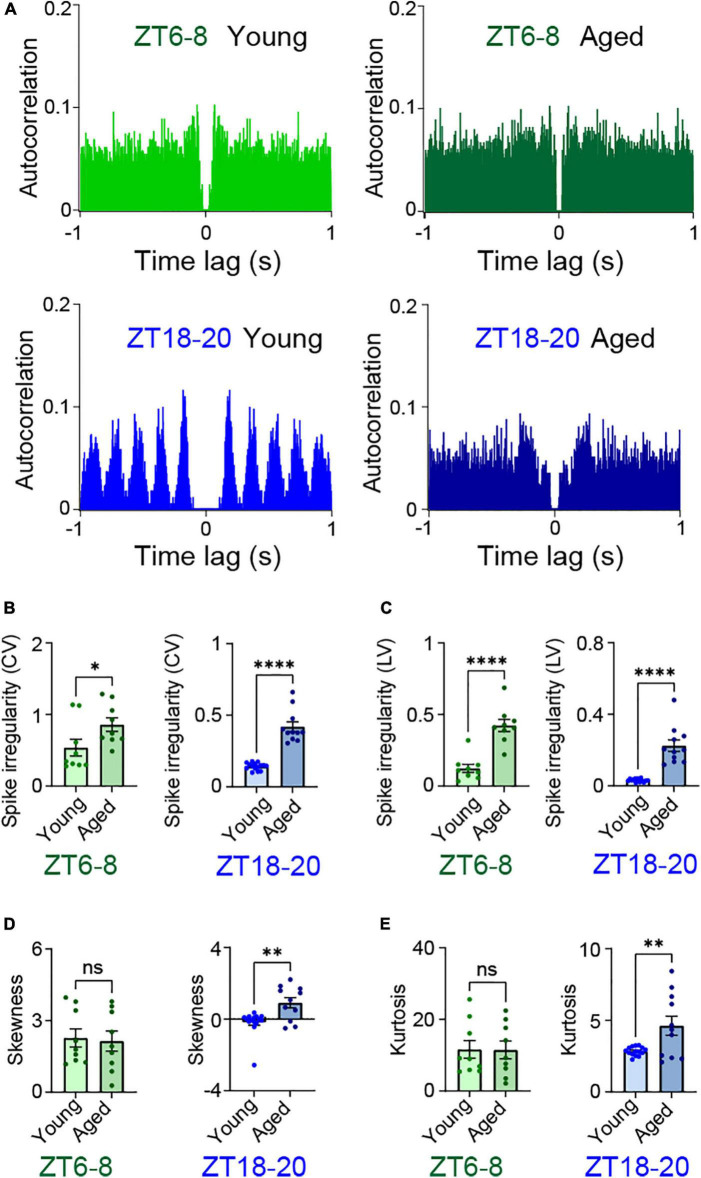
Aging altered spike patterns during spontaneous activity. **(A)** Autocorrelation functions of spontaneous firing in young and aged DN1p neurons at ZT6–8 and ZT18–20. **(B–E)** Statistical parameters related to temporal structural regularity of spike trains in young and aged DN1p neurons at ZT6–8 and ZT18–20 (ZT6–8, young: *n* = 9, aged: *n* = 9; ZT18–20, young: *n* = 15, aged: *n* = 11; same data used in Figure 1). Spike irregularity shown as the ratio of standard deviation to the mean of ISIs (coefficient of variation, CV) **(B)**, spike irregularity shown as the dispersion of two adjacent ISIs (local variation, LV) **(C)**, degree of asymmetry of the probability distribution of ISIs (skewness) **(D)**, and measure of the sharpness of the probability distribution of ISIs (kurtosis) **(E)**. *p < 0.05, ^**^p < 0.01, and ^****^p < 0.0001 based on *t*-tests.

The DN1p circadian clock adjusts ionic flux in a time-dependent manner to alter its own neural activity. Also, spontaneous activity in DN1p neurons shows the clock-generated formation of unique temporal spiking patterns, defined by the second-order temporal structure of ISIs. Because we found remarkable age-associated changes in the pattern of DN1p neural activity, we analyzed how aging alters the temporal structure of ISIs. First, we quantified the autocorrelation function based on spike timing. The structured autocorrelation function only occurred in DN1p neurons of young flies at ZT18–20 (Figure 2A). Next, we examined the relationship between spike irregularity and aging. The CV of ISIs was used as a global metric of spike irregularity statistics (Figure 2B), the LV of adjacent ISIs was used as a local metric of spike irregularity statistics (Figure 2C). In both cases, the temporal structural irregularity was significantly greater in aged vs. young DN1p neurons at both ZT6–8 and ZT18–20.

To define the shape of the ISI histogram (Supplementary Figure 1), we quantified the degrees of skewness and kurtosis. The spiking patterns in aged DN1p neurons showed significantly different skewness (Figure 2D) and kurtosis (Figure 2E) in young vs. aged DN1p neurons at ZT18-20, but not ZT6-8. We assessed how activity fluctuations differ in the frequency domain with continuous wavelet transform. We found that aged DN1p neurons showed an altered spectral power structure (Supplementary Figure 2). These results indicate that aging dramatically alters the temporal structure of spontaneous firing patterns of DN1p neurons, especially during nighttime.

### Age Is Associated With Altered Membrane Potential Responses in DN1p Neurons

**FIGURE 3:**
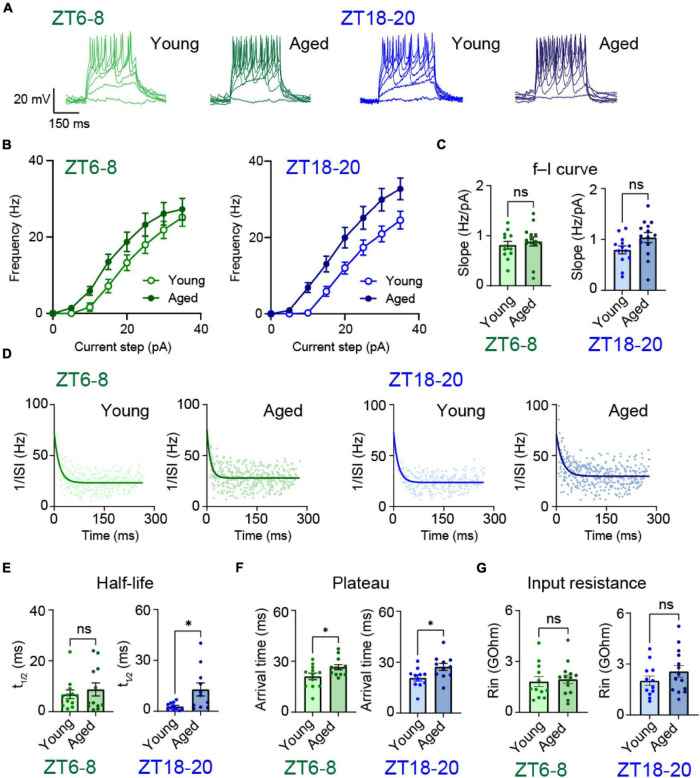
Aging altered membrane potential responses elicited by current injections in DN1p neurons. **(A)** Representative membrane potential traces of spontaneous firing in young and aged DN1p neurons at ZT6–8 and ZT18–20. **(B–E)** Mean firing rate vs. injected current **(B)**, slope factor obtained from linear regression **(C)**, instantaneous spike frequency vs. occurrence time showing spike frequency adaptation **(D)**, half-life spike **(E)** and plateau **(F)** obtained from curve fitting, and input resistance **(G)** of young and aged DN1p neurons at ZT6–8 and ZT18–20 (ZT6–8, young: *n* = 12, aged: *n* = 14 ZT18–20, young: *n* = 12, aged: *n* = 14). *p < 0.05 based on *t*-tests.

To further delineate biophysical parameters, we assessed age-associated changes in intrinsic membrane properties by measuring the membrane potential dynamics of DN1p neurons in response to current injections (Figure 3A). The frequency-current relationships (*f-I* curve) showed greater excitability in aged DN1p neurons vs. young DN1p neurons (Figure 3B). However, based on the linear regression slope, the *f-I* curve did not significantly differ between young and aged flies, regardless of the circadian timing (Figure 3C).

Next, we quantified the degree of spike frequency adaptation by fitting instantaneous spike frequency with a monoexponential relation (Figure 3D). When sustaining a depolarizing current injection, all experimental conditions showed a spike frequency adaptation. We also compared the relation, half-life (Figure 3E), and plateau (Figure 3F) variables of the fitting. Although the half-life did not significantly differ between young and aged DN1p at ZT6-8, it was significantly less in young vs. aged DN1p neurons at ZT18-20 (young at ZT6-8: 6.9 ± 1.7 ms, aged at ZT6-8: 8.7 ± 2.5 ms, young at ZT18-20: 2.8 ± 0.67 ms, aged at ZT18-20: 12.8 ± 3.79 ms) (Figure 3E). These findings indicate that aging weakened the adaptation strength. The plateau significantly differed between young and aged DN1p neurons at both ZT6-8 and ZT18-20 (young at ZT6-8: 21.03 ± 1.92 ms, aged at ZT6-8: 26.5 ± 1.56 ms, young at ZT18-20: 20.7 ± 1.76 ms, aged at ZT18-20: 27.1 ± 2.1 ms) (Figure 3F), suggesting that aged DN1p neurons have a lower baseline of depolarization sensitivity.

We also measured input resistance as a passive membrane property. The input resistance did not significantly differ between young and aged DN1p neurons at both ZT6-8 and ZT18-20 (Figure 3G).

### Age Is Associated With Reduced Synaptic Inputs in DN1p Neurons

**FIGURE 4:**
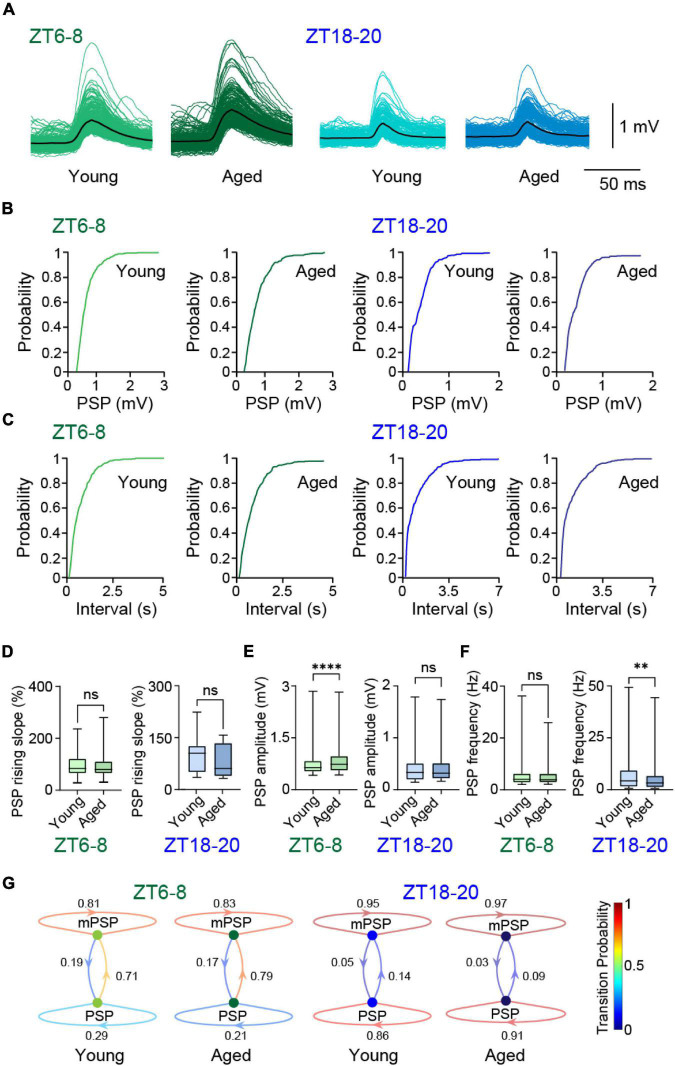
Aging altered synaptic inputs in DN1p neurons. **(A)** Superimposed membrane potential traces based on postsynaptic potential (PSP) in young and aged DN1p neurons at ZT6–8 and ZT18–20. **(B–E)** Cumulative probability distributions of PSP amplitude **(B)** and inter-event interval values **(C)**; quantification of relative rising slope of PSPs **(D)**, PSPs amplitude **(E)**, and PSPs frequency **(F)**; and discrete-time Markov chain showing transition probability between estimated mPSP and PSP **(G)** in young and aged DN1p neurons at ZT6–8 and ZT18–20 (ZT6–8, young: *n* = 294 PSPs from *N* = 3 DN1ps, aged: *n* = 384 PSPs from *N* = 3 DN1p neurons; ZT-18–20, young: *n* = 333 PSPs from *N* = 3 DN1p neurons, aged: *n* = 391 PSPs from *N* = 4 DN1p neurons). ^**^p < 0.01 and ^****^p < 0.0001 based on Mann-Whitney *U*-tests.

To test whether synaptic input contributes to the age-related changes of spike patterns in DN1p neurons, we used quartz glass with a sharp electrode to measure spontaneous synaptic input. While constantly injecting a hyperpolarizing current from a sharp electrode, spontaneous postsynaptic potential (PSP) readily occurred (Figure 4A). Individual PSP epochs were sorted to show the cumulative probability of individual amplitude (Figure 4B) and inter-event interval (Figure 4C). These PSP events were quantified in functional biophysical parameters. Whereas the relative rising slope of the PSPs did not significantly differ between young and aged DN1p neurons at both ZT6-8 and ZT18-20 (Figure 4D), the PSP amplitude was significantly greater in aged DN1p neurons at ZT6-8 but not at ZT18-20 (Figure 4E). In contrast, the PSP frequency was significantly lower in aged samples at ZT18-20 but not at ZT6-8 (Figure 4F).

We also assessed if aging alters state transition probability in the temporal structure of synaptic input sequences. To do this, we created a discrete-time Markov chain showing the state transition between estimated miniature PSP (mPSP) and PSP (Figure 4G). The state transition probability increased from PSP to mPSP in young and aged DN1p neurons at ZT6-8 but not at ZT18–20. These results indicate that aging leads to directional changes in synaptic inputs of DN1p neurons between daytime and nighttime. These changes should contribute to age-related changes in DN1p spike patterns by interacting with intrinsic membrane properties.

### Age Alters the Localization of Slowpoke Binding Protein and Na^+^/K^+^ ATPase β Subunit in DN1p Neurons

**FIGURE 5:**
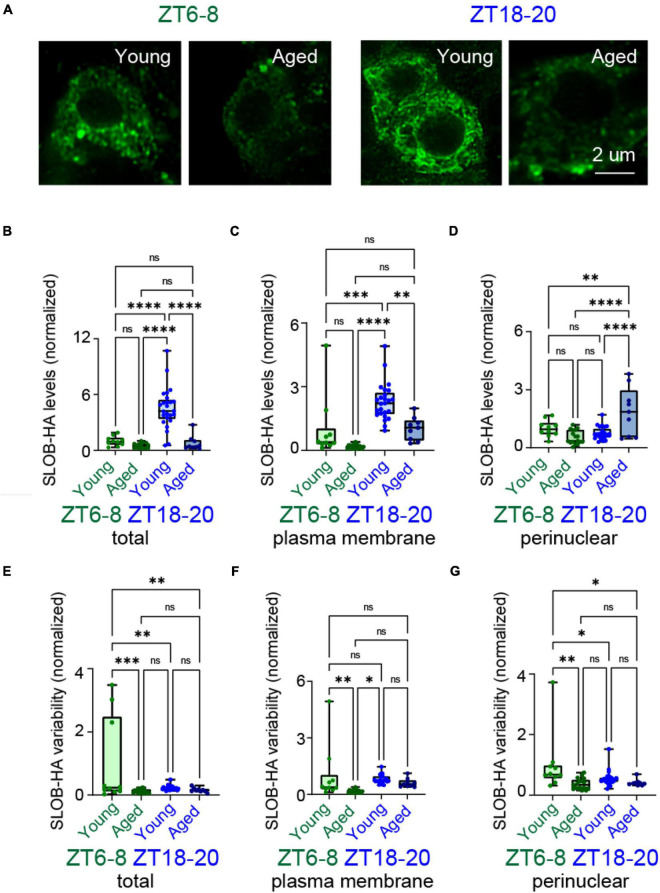
Aging changed time-dependent localization patterns of SLOB in DN1p neurons. **(A)** Anti-HA (green) immunostaining of DN1p neurons in young and aged wake-GAL4 > UAS-SLOB-HA flies at ZT6–8 and ZT18–20. Scale bar indicates 2 μm. **(B–D)** Quantification of total **(B)**, plasma membrane **(C)**, and perinuclear **(D)** SLOB-HA levels in young vs. aged DN1p neurons at ZT6–8 and ZT18–20. **(E–G)** Quantification of distribution variability for total **(E)**, plasma membrane **(F)**, and perinuclear **(G)** SLOB-HA signals in young vs. aged DN1p neurons at ZT6–8 and ZT18–20 (ZT6–8, young: *n* = 10, aged: *n* = 17; ZT18–20, young: *n* = 25, aged: *n* = 9). *p < 0.05, ^**^p < 0.01, ^***^p < 0.001, and ^****^p < 0.0001 based on two-way ANOVA with multiple comparisons.

**FIGURE 6:**
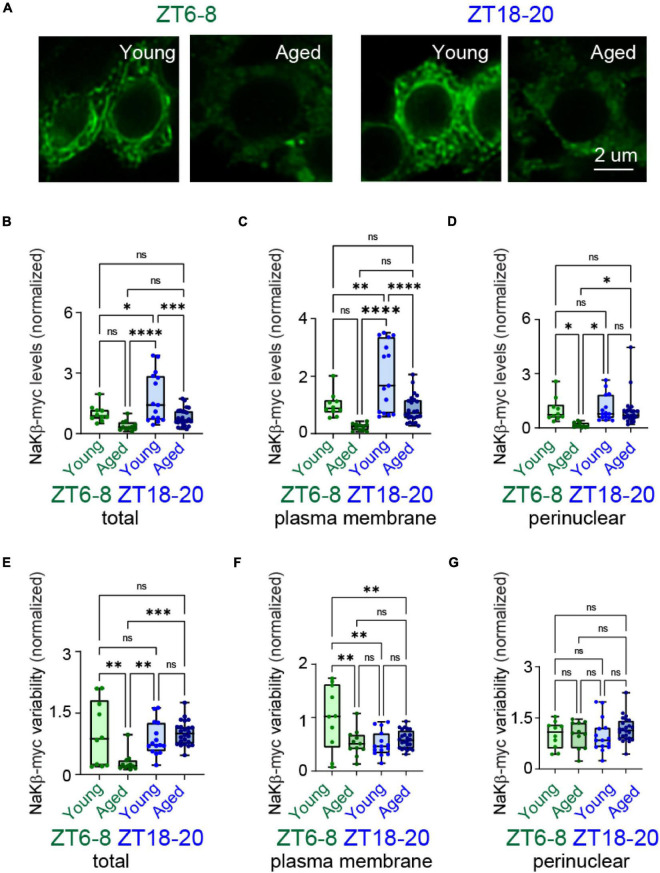
Aging changed time-dependent localization patterns of NaKβ in DN1p neurons. **(A)** Anti-myc (green) immunostaining of DN1p neurons in young and aged wake-GAL4 > UAS-NaKβ-myc flies at ZT6–8 and ZT18–20. Scale bar indicates 2 μm. **(B–D)** Quantification of total **(B)**, plasma membrane **(C)**, and perinuclear **(D)** NaKβ-myc levels in young vs. aged DN1p neurons at ZT6–8 and ZT18–20. **(E–G)** Quantification of distribution variability for total **(E)**, plasma membrane **(F)**, and perinuclear **(G)** NaKβ-myc signals in young vs. aged DN1p neurons at ZT6–8 and ZT18–20 (ZT6–8, young: *n* = 10, aged: *n* = 11; ZT18–20, young: *n* = 15, aged: *n* = 24). *p < 0.05, ^**^p < 0.01, ^***^p < 0.001, and ^****^p < 0.0001 based on two-way ANOVA with multiple comparisons.

Slowpoke binding protein (SLOB) and Na^+^/K^+^ ATPase β subunit (NaKβ) contribute to membrane potential dynamics in DN1p neurons (Tabuchi et al., 2018). Moreover, these molecules showed circadian-dependent changes in their subcellular localization when regulated by clock and wake signaling in circadian neuronal networks. We hypothesized that aging disrupts the localization of SLOB and NaKβ, leading to changes in electrophysiological properties. To determine if the subcellular localization of these factors changes with age, we used transgenic flies expressing HA-tagged SLOB (SLOB-HA) and myc-tagged NaKβ (NaKβ-myc) in their DN1p neurons. Because DN1p neurons are small and have limited accessibility for quantification, we used stimulated emission depletion microscopy to quantify the subcellular patterns in these neurons. With this approach, we readily observed the subcellular patterns of SLOB-HA and NaKβ-myc (Figures 5A, 6A). We found that total SLOB expression was significantly greater in young vs. aged DN1p neurons at ZT18-20 but not at ZT6-8 (Figure 5B). This age-related effect was remarkably greater at the plasma membrane (Figure 5C). Additionally, we found that SLOB showed a significant increase in the perinuclear region in aged flies compared to young flies (Figure 5D), suggesting a possible mistargeting to membrane trafficking pathways. We also quantified the heterogeneity of SLOB expression within DN1p neurons based on signal variability (Figure 5E). We found that the signal variability was remarkably greater in young vs. aged DN1p neurons at ZT6-8, but not significantly different at ZT18-20. Subcellular variability also did not differ between the plasma membrane (Figure 5F) and the perinuclear region (Figure 5G).

We also assessed the expression patterns of myc-NaKβ in DN1p neurons (Figure 6A). NaKβ expression was significantly greater in young vs. aged DN1p neurons at ZT18-20 but not at ZT6-8 (Figure 6B). Similar to SLOB, NaKβ expression was remarkably greater at the plasma membrane (Figure 6C) but not at the perinuclear region (Figure 6D) in aged DN1p neurons. We also analyzed changes in the signal variability of NaKβ. Similar to SLOB, the signal variability of NaKβ was most heterogeneous in young DN1p neurons at ZT6-8 and significantly less heterogeneous in aged DN1p neurons (Figure 6E). The signal variability did not significantly differ in the perinuclear compartments under any experimental condition (Figure 6G), suggesting that such heterogeneity was largely driven by differences in the plasma membrane compartments (Figure 6F).

## Discussion

In this study, we compared electrophysiological properties between young and aged DN1p neurons and found age-associated changes in both intrinsic membrane properties and extrinsic synaptic inputs. Specifically, we found that aged DN1p neurons have drastically unstructured spike patterns of spontaneous activity during nighttime. These unstructured spike patterns may result from changes in biophysical parameters related to spike frequency adaptation and synaptic input properties. Also, the molecules SLOB and NaKβ showed circadian cycling in their subcellular localization, such that aging reduced their levels and heterogenous distribution. Interestingly, these aging effects were both circadian time-dependent and -independent. This relationship suggests that multiple age-associated factors contribute to different aspects (some of which may come from circadian clock signaling or be related to more generalized cellular signaling) that occur simultaneously. The most remarkable difference in aged DN1p neurons was functional parameters related to activity patterns.

Several reports have investigated the activity patterns of clock neurons during aging (Buijink and Michel, 2021). For example, in aged hamsters, SCN neuronal activity deteriorated (Watanabe et al., 1995). Also, in SCN neurons of aging mice, daily rhythmic changes in the mean firing rate were reduced (Nakamura et al., 2011). These studies were based on broad measurements in cell populations (e.g., cell-type specificity was relatively unclear) of the SCN. They also performed recording over the course of the days, so they could not detect the precise regularity of pattern changes based on ISI variability. In this study, we were able to detect these changes. We also delineated a possible correlation between ISIs temporal sequence, intrinsic membrane properties, and synaptic properties, all of which were altered by aging. These findings were first achieved by focusing on a specific circadian time to analyze membrane potential dynamics of very rigorous cell-type identities available from *Drosophila* circadian networks. Also, we found that several key biophysical parameters, such as AHP and spike onset depolarization duration, account for unstructured spike patterns in aged DN1p neurons.

Our results show that aging alters intrinsic membrane properties in DN1p neurons during spontaneous activity at nighttime but not daytime. However, aging altered spike frequency adaptation at both daytime and nighttime. The difference between these results may derive from increased depolarization sensitivity during aging. During daytime, the circadian network, including DN1p neurons, tends to receive a constant depolarization from environmental light inputs. As a result, frequency adaptation is likely a default mode that decreases depolarization sensitivity. On the other hand, aging alters the dynamic relationships between spontaneous activity and evoked excitability by increasing a biophysical space for accepting more spike frequency adaptation. Thus, this age-dependent process could be coupled with changes in spiking patterns during nighttime.

Dynamic relationships between spontaneous and evoked electrophysiological activity have been proposed in technical contexts (Wainio-Theberge et al., 2021). However, altered biophysical parameters may contribute to evoked electrophysiological activity during daytime that has a greater effect on spontaneous activity patterns during nighttime vs. daytime. Recent studies conducting electrophysiological recordings of the circadian network in *Drosophila* and mice showed a number of age-related changes. Our results in *Drosophila* DN1p neurons recapitulated their findings and brought new insight. We showed that aged DN1p neurons have significantly reduced PSP frequency in aged DN1p neurons at ZT18-20, which may result from age-related changes in neuronal excitability in l-LNv neurons (Curran et al., 2019).

SLOB and NaKβ are primarily responsible for regulating activity patterns in DN1p neurons (Tabuchi et al., 2018). Thus, we assessed age-related changes in their subcellular organization patterns during circadian cycling by expressing SLOB-HA and NaKβ-myc in young and aged DN1p neurons. In young flies, both SLOB and NaKβ localized at the plasma membrane of DN1p neurons at higher levels during nighttime than daytime. On the other hand, subcellular localization of SLOB and NaKβ was disrupted in aged DN1p neurons. These molecules showed relatively homogeneous expression in aged DN1p neurons vs. more heterogeneous expression in young DN1p neurons, suggesting that aging may dysregulate localization of SLOB and NaKβ. A similar result occurred in non-neuronal cells: heterogeneous signal distribution was lost with aging (Ito et al., 2010; van Deventer et al., 2015). These findings support that aging influences the molecular organization of precisely structured activity patterns in the circadian network, leading to age-associated decreases in sleep quality. If the mistargeting of SLOB and NaKβ can be addressed by identifying factors mediating their membrane trafficking to DN1p neurons, the overexpression of SLOB and NaKβ may be useful in improving sleep quality in old flies.

In general, precisely structured activity patterns in the brain play critical functions throughout life. These functions range from synapse formation, pruning, and network wiring during development (Nakashima et al., 2019) to processing performance for sensory perception encoding (Wehr and Laurent, 1996; Smith and Lewicki, 2005; Cassenaer and Laurent, 2007; Gollisch and Meister, 2008), cognition (Skaggs et al., 1996), and memory processing (Louie and Wilson, 2001; Marshall et al., 2006; Ego-Stengel and Wilson, 2010) post-maturation. In circadian networks, structured activity patterns optimize processing performance to regulate sleep/arousal states in a time-dependent manner. With aging, these structured patterns are disrupted, causing sleep fragmentation and cognitive decline, which may be resulting in neurodegenerative diseases. In the *Drosophila* circadian network, environmental light information is received with both visual input and intrinsic photosensitivity and affects circadian clock rhythms (Helfrich-Forster et al., 2001; Klarsfeld et al., 2004). Interestingly, several studies have shown possible relationships between light/clock signaling and aging in *Drosophila* (Rakshit and Giebultowicz, 2013; Nash et al., 2019) and humans (Daneault et al., 2016; Nag, 2021). It is worth noting that our data was acquired under light/dark cycle, and data acquisitions under constant darkness would be helpful for understanding how age-related changes in neural activity patterns in DN1p neurons can be signified by environmental light information.

In summary, this study shows that aging disrupts the subcellular organization of SLOB and NaKβ to alter activity patterns in DN1p clock neurons of *Drosophila*. These findings support the emerging view that age-associated electrophysiological changes in both intrinsic membrane properties and synaptic transmission alter the structure of brain activity patterns. As the impacts of circadian dysfunction on aging are essentially conserved from *Drosophila* to humans (Mhatre et al., 2014; De Nobrega and Lyons, 2020) and age-related physiological changes in the circadian network is a hallmark of age-associated pathologies including neurodegenerative diseases (Hood and Amir, 2017), understanding these mechanisms could provide targets for the development of therapeutics for age-related neurodegenerative diseases.

## Data Availability Statement

The raw data supporting the conclusions of this article will be made available by the authors, without undue reservation.

## Author Contributions

MT designed the study. DN, AH, YZ, SD, AP, and MT performed the experiments and data analysis. MT wrote the manuscript with input from DN, AH, YZ, SD, and AP. All authors contributed to the article and approved the submitted version.

## Conflict of Interest

The authors declare that the research was conducted in the absence of any commercial or financial relationships that could be construed as a potential conflict of interest.

## Publisher’s Note

All claims expressed in this article are solely those of the authors and do not necessarily represent those of their affiliated organizations, or those of the publisher, the editors and the reviewers. Any product that may be evaluated in this article, or claim that may be made by its manufacturer, is not guaranteed or endorsed by the publisher.

## Footnotes

1
https://www.janelia.org/open-science/wavesurfer

## Supplementary Material

Supplementary Figure 1 Histogram of the distribution of interspike intervals during spontaneous firing activity in DN1p neurons (same data used in Figures 1, 2).

Supplementary Figure 2 Continuous wavelet transform heatmaps showing scaled and normalized oscillatory amplitude according to frequency and time (same data used in Figures 1, 2).
