# Supplementary figures and images for: Age-Related Unstructured Spike Patterns and Molecular Localization in Drosophila Circadian Neurons
Source: Front Physiol. 2022 Mar 9;13:845236. doi: 10.3389/fphys.2022.845236 (PMC8959858; doi:10.3389/fphys.2022.845236)

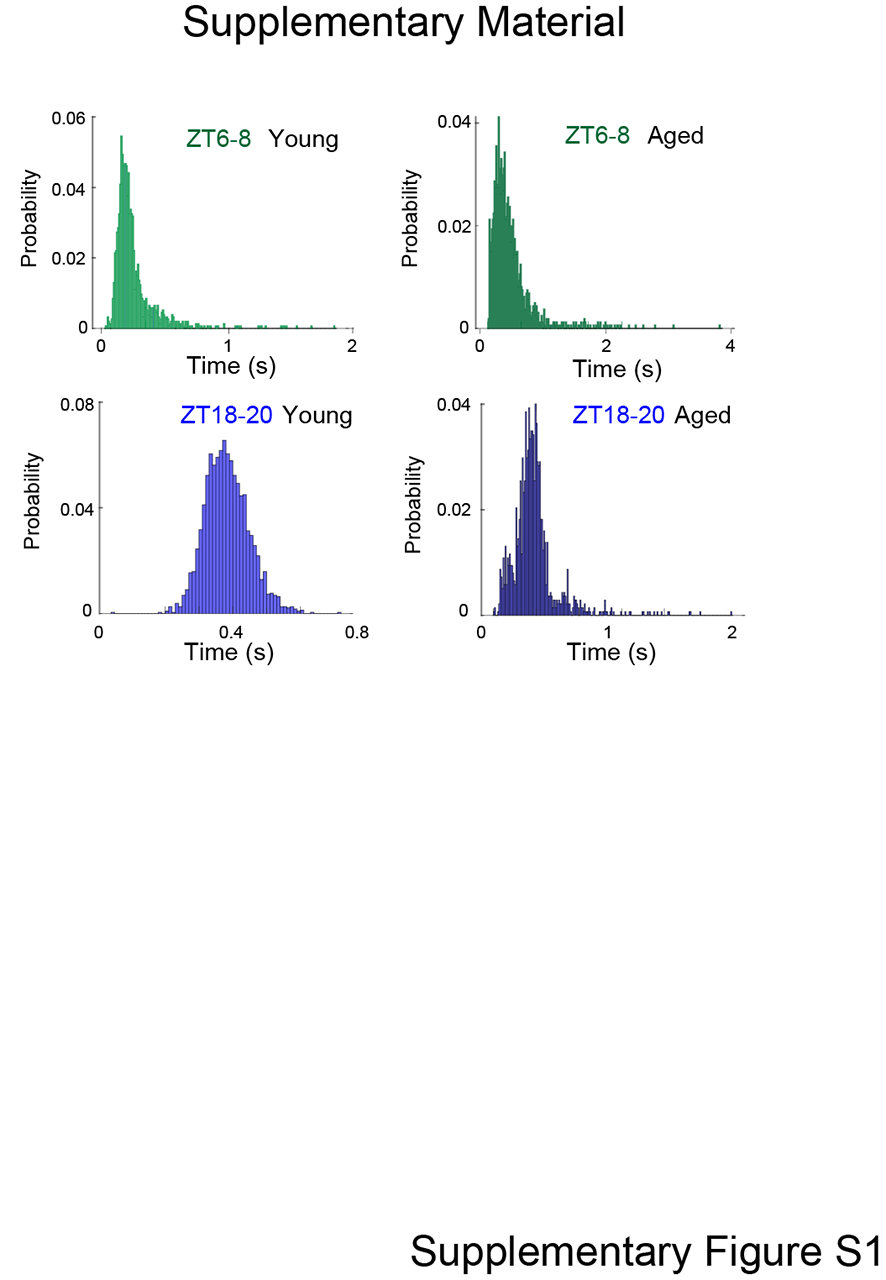

Supplement: Supplementary Figure 1 — Histogram of the distribution of interspike intervals during spontaneous firing activity in DN1p neurons (same data used in Figures 1, 2). [file Image_1.tif]

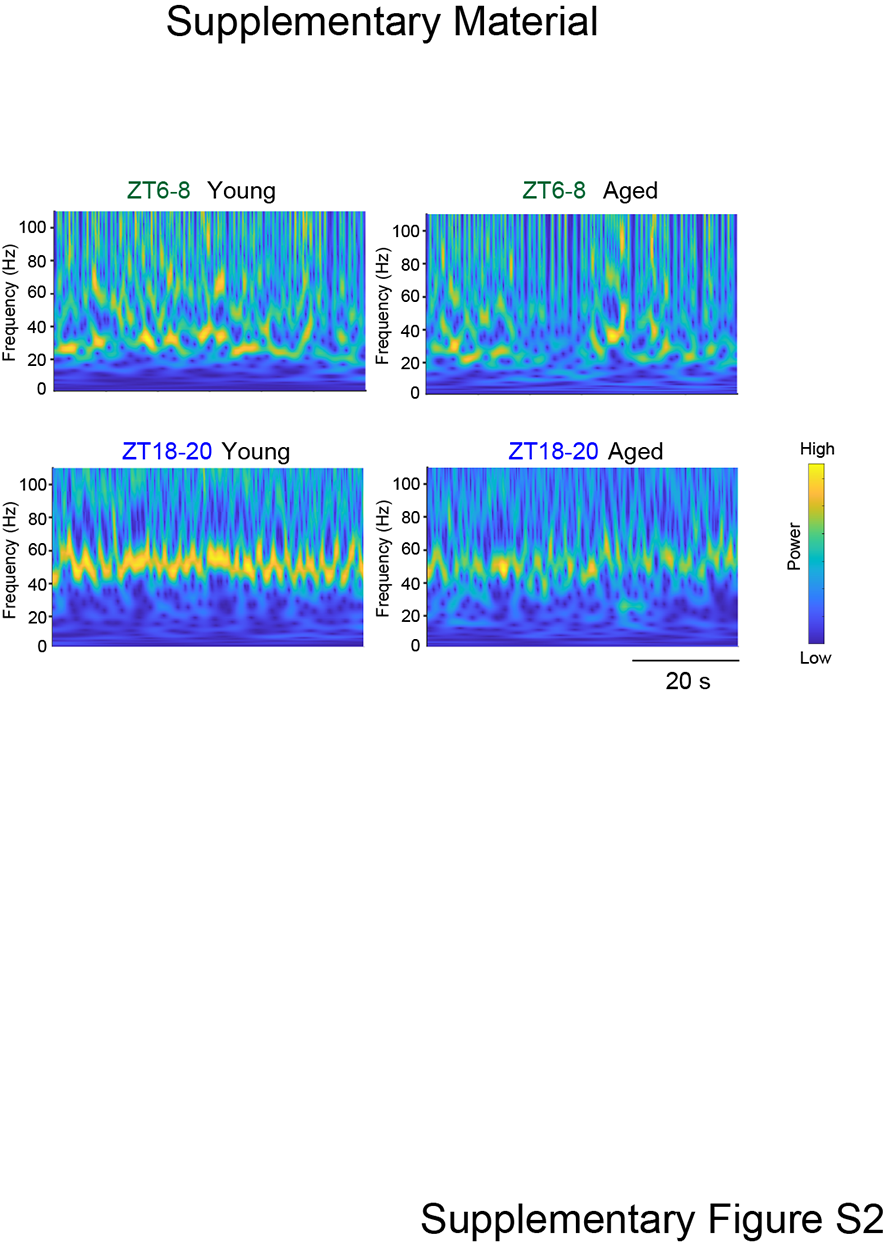

Supplement: Supplementary Figure 2 — Continuous wavelet transform heatmaps showing scaled and normalized oscillatory amplitude according to frequency and time (same data used in Figures 1, 2). [file Image_2.tif]
